# Fe-doped carbon dots: a novel biocompatible nanoplatform for multi-level cancer therapy

**DOI:** 10.1186/s12951-023-02194-6

**Published:** 2023-11-17

**Authors:** Mingxi Yang, Haiqiu Li, Xinchen Liu, Lei Huang, Boya Zhang, Kexuan Liu, Wangni Xie, Jing Cui, Daowei Li, Laijin Lu, Hongchen Sun, Bai Yang

**Affiliations:** 1 Joint Laboratory of Opto-Functional Theranostics in Medicine and Chemistry, The First Hospital of Jilin University, Changchun, 130021 People’s Republic of China; 2 Jilin Provincial Key Laboratory of Tooth Development and Bone Remodeling, Hospital of Stomatology, Jilin University, Changchun, 130021 People’s Republic of China; 3 Department of Hand and Podiatric Surgery, Orthopedics Center, The First Hospital of Jilin University, Jilin University, Changchun, 130031 People’s Republic of China

**Keywords:** Carbon dots, Fe-doping, Anti-tumor, Apoptosis, Antitumoral macrophages, Epithelial-mesenchymal transition

## Abstract

**Background:**

Tumor treatment still remains a clinical challenge, requiring the development of biocompatible and efficient anti-tumor nanodrugs. Carbon dots (CDs) has become promising nanomedicines for cancer therapy due to its low cytotoxicity and easy customization.

**Results:**

Herein, we introduced a novel type of “green” nanodrug for multi-level cancer therapy utilizing Fe-doped carbon dots (Fe-CDs) derived from iron nutrient supplement. With no requirement for target moieties or external stimuli, the sole intravenous administration of Fe-CDs demonstrated unexpected anti-tumor activity, completely suppressing tumor growth in mice. Continuous administration of Fe-CDs for several weeks showed no toxic effects in vivo, highlighting its exceptional biocompatibility. The as-synthesized Fe-CDs could selectively induce tumor cells apoptosis by BAX/Caspase 9/Caspase 3/PARP signal pathways and activate antitumoral macrophages by inhibiting the IL-10/Arg-1 axis, contributing to its significant tumor immunotherapy effect. Additionally, the epithelial-mesenchymal transition (EMT) process was inhibited under the treatment of Fe-CDs by MAPK/Snail pathways, indicating the capacity of Fe-CDs to inhibit tumor recurrence and metastasis.

**Conclusions:**

A three-level tumor treatment strategy from direct killing to activating immunity to inhibiting metastasis was achieved based on “green” Fe-CDs. Our findings reveal the broad clinical potential of Fe-CDs as a novel candidate for anti-tumor nanodrugs and nanoplatform.

**Supplementary Information:**

The online version contains supplementary material available at 10.1186/s12951-023-02194-6.

## Introduction

Although various treatment modalities, such as chemotherapy [1], radiation therapy [2] and phototherapy [3, 4], have been explored, the prognosis of tumors still remains poor and is accompanied by severe systemic side effects. The therapeutic effects, however, are often seriously diminished within the immunosuppressive tumor microenvironment (TME) [5, 6]. The phenotype polarization of tumor-associated macrophages (TAMs) is one of the main reasons for the immune inactivation of cytotoxic T cell, leading to the failure of tumor immunotherapy [7, 8]. Even if the tumor is effectively suppressed in the first treatment, the metastases and recurrences of tumor are far more severe than the primary tumor, resulting in the majority of deaths from cancer. The process of epithelial-mesenchymal transition (EMT) is considered to primarily assist tumor cells in evading treatment and spreading throughout the body [9, 10]. Thus, it suggests that single-level intervention in a certain process during tumor growth is not enough to overcome cancer, which puts forward higher requirements for treatment methods and anti-tumor drugs.

Over the past decades, exciting progress has been achieved in nanomaterials with enhanced capacities to kill tumor and reinvigorate the immunity within TME [11, 12]. In order to achieve satisfactory therapeutic effect, it is generally necessary to integrate several components into the nanocarriers and external stimuli applied during therapy, resulting in cumbersome procedure and poor reproducibility, which might lead to unpredictable treatment outcomes and limitations of clinical translation. Recently, single-component nanoparticles (SNPs) with all-in-one functions have been proved to play an important role in the new campaign of anti-tumor, due to their advantages of simple composition, excellent biocompatibility, and high stability [13]. Moreover, utilizing SNPs with multiple roles can immensely reduce the possible safety risks to the organisms, which is in line with contemporary development and clinical transformation of nanomedicine. However, there are still few reports on artificially synthesized SNPs with multi-directional anti-tumor ability of tumorigenesis and metastasis, resulting in the slack development of SNPs that are promising for clinical application.

Since the VIII group heavy metal complexes represented by Pt(IV), Ru(II), and Rh(III) have been used in clinical treatment of cancer [14], metal doping strategies are also introduced to develop multi-functional materials with enhanced anti-tumor efficacy. In order to avoid potential toxicity issue, several works have reported various transition metal ions-doped nanomaterials, e.g., Fe^2+^ [15], Cu^+^ [16], and Mn^2+^ [17], which endow the nanoparticles with chemodynamic therapy (CDT) function by mediating Fenton or Fenton-like reaction, inducing metal ions-stimulated immunogenic tumor cell death and activate immune cells [18]. Due to the important status of iron metabolism in TME and the “iron addiction effect” of tumor cells [19], Fe-containing proteins, i.e., hemoglobin [20] and ferritin [21], and Fe-doping nanomaterials, i.e., iron-coordinated polyphenol/amino acid nanoassembly drugs [22–24], have been employed to elevate the iron level in tumor cells to enhance ferroptosis. However, they still face with shortcomings such as relatively large particle size, poor water solubility and stability, low uptake efficiency, and combination of chemotherapy or phototherapy is required to achieve complete tumor elimination. Carbon dots (CDs), as an emerging carbon-based nanomaterials, has revealed great application prospects in the field of theranostic and drug delivery, due to its low toxicity, strong penetrating ability, ease of modification and stable physicochemical properties [25–28]. In this regard, the previously reported CDs used for anti-tumor were generally combined with photothermal [29], photodynamic therapy [30], chemotherapeutic drugs [31, 32] or loaded with mRNAs [33]. To the best of our knowledge, there is no direct application of CDs that can inhibit the growth of tumors while also regulating immunity and preventing metastasis.

**Scheme 1:**
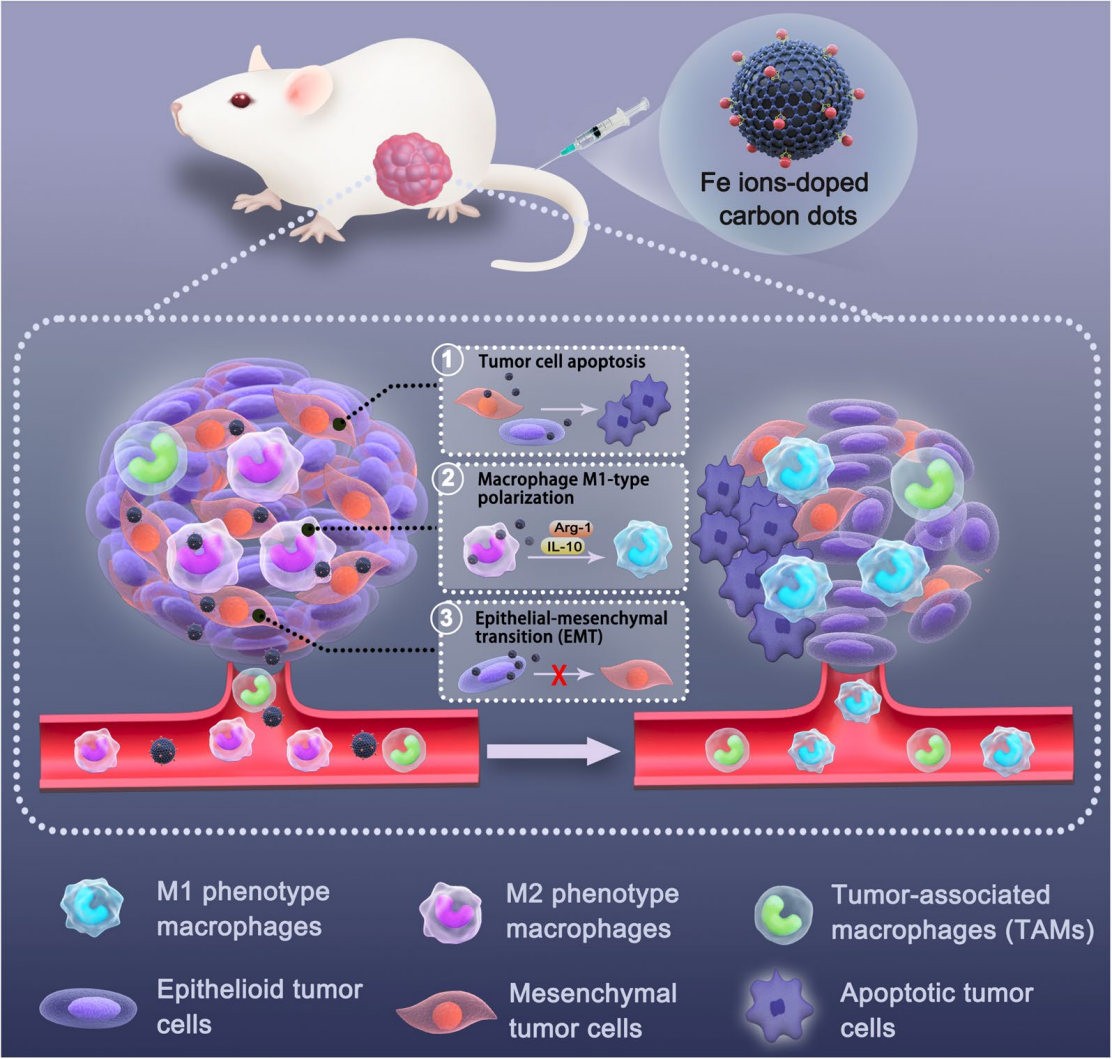
Schematic illustration of anti-tumor effect of Fe-CDs through multi-level remodeling the tumor microenvironment: (1) inducing tumor cells apoptosis, (2) activating antitumoral macrophages immunity, and (3) inhibiting epithelial-mesenchymal transition (EMT)

Herein, for the first time, a three-level anti-tumor therapeutic strategy (inducing apoptosis, activating immunity, and preventing metastasis) is proposed based on a novel type of “green” Fe-doped CDs (Fe-CDs) (Scheme 1), which is derived from a iron fortifier. The tumor growth can be significantly impeded via intravenous administration of Fe-CDs, and the tumor even completely disappeared without introducing other drugs and external stimuli. Mechanistic studies reveal that Fe-CDs can selectively promote tumor cell apoptosis through regulating PARP/Caspase 3/Caspase 9/BAX proteins, and activate antitumoral macrophages by inhibiting the IL-10/Arg-1 axis, which contributed to the superior anti-tumor performance of Fe-CDs. Moreover, Fe-CDs effectively suppressed EMT process of tumor cells via MAPK/Snail signal pathways, further preventing tumor metastasis and recurrence. In addition, long-term continuous administration of Fe-CDs did not significantly affect the body weight or important organs of mice, indicating its superior biocompatibility. This simply synthesized bio-friendly Fe-CDs are promising as candidates for the next generation of anti-tumor nanoplatform with bright clinical application prospects.

## Methods

### Materials

Ferrous gluconate dihydrate (FG, C_12_H_22_O_14_Fe·2H_2_O) was purchased from Macklin Reagent Inc. (Shanghai, China), and used directly without further purification. Sulfo-Cyanine 5.5 (Cy5.5) was purchased from ToYongBio Tech. Inc. (Shanghai, China). Phosphate buffered saline powder (PBS) was purchased from Monad, Biotech Co., Ltd (Suzhou, China). Dulbecco’s Modified Eagle’s Medium (DMEM), Leibovitz’s L-15 medium, Roswell Park Memorial Institute medium (RPMI) were purchased from GIBCO BRL (Grand Island, NY, USA). Foetal bovine serum (FBS) was purchased from Clark Bioscience (Australia). Trypsin was purchased from NCM Biotech (Suzhou, China). Prussian blue was purchased from Solarbio Science & Technology Co., Ltd (Beijing, China). Deionized water was used throughout the whole experiments.

### Synthesis of Fe-CDs

The Fe-CDs was synthesized by one-step hydrothermal-assisted carbonization reaction according to our previous work with a slight modification [34]. Briefly, 2 mmol FG was dissolved in 40 mL deionized water, and completely dissolved by sonication treatment. Then the solution was transferred to a Teflon-lined autoclave and enclosed in a stainless steel reactor shell, followed by heating in an oven at 180 °C for 4 h. After cooling to room temperature, the dark-brown reaction solution was filtered through a 0.22 μm polyethersulfone membrane to remove the large aggregate. Subsequently, the solution was dialyzed with a 500–1000 Da dialysis tube against water for 24 h to remove small fragments and raw materials. The powder of Fe-CDs could be finally obtained by lyophilization. The G-CDs without Fe doping was synthesized from glucolactone through the same reaction procedure as Fe-CDs, which served as a Fe-free CDs control.

### Characterization of Fe-CDs

The high-resolution transmission electron microscope (TEM) images were recorded on JEOL JEM-2100F. The elemental analysis was performed on the energy dispersive X-ray spectrometer (EDS) integrated on TEM. The photoluminescence (PL) and ultraviolet–visible (UV–vis) absorption spectra were measured on Shimadzu RF-5301 PC and 3100 UV–Vis spectrophotometer, respectively. The Fourier transform infrared (FTIR) spectra was monitored on Nicolet Avatar 360 FTIR spectrophotometer. The X-ray diffraction (XRD) pattern was obtained on PANalytical B.V. Empyrean small-angle diffractometer. The X-ray photoelectron spectroscopy (XPS) analyzes were acquired from ESCALAB 250 spectrometer with a mono X-ray source Al Kα excitation (1486.6 eV). The metal content was tested by Agilent 725 inductively coupled plasma spectrometer (ICP).

### Cell culture

Human triple-negative breast cancer cells (MDA-MB-231), mouse breast cancer cells (4T1), human umbilical vein endothelial cells (EA.hy926), and mouse mononuclear macrophage leukemia cells (RAW 264.7) were purchased from the Cell Bank of the Shanghai Institute of Biological Sciences (Shanghai, China) and cultured in L-15 medium, RPMI medium, and DMEM medium. Human dental pulp stem cells (hDPSCs) were purchased from Procell Life Technology Co., LTD (Wuhan, China) and cultured in complete medium for human dental pulp stem cells (CM-H231, Procell, China). All media were supplemented with 10% FBS. All cell lines, except for triple-negative breast cancer cells, were cultured in a humidified environment at 37 °C and 5% CO_2_, with medium changed daily. The cells were typically passaged using trypsin, and were isolated from culture containers for subculture and identified by morphology. All cell lines were negative by mycoplasma testing.

### Cytotoxicity assay

Cytotoxicity of Fe-CDs and FeG was determined by MTT assay (C0009S, Beyotime, China). Human triple-negative breast cancer cells (MDA-MB-231, provided by Chinese Academy of Sciences Cell Bank) were seeded at an initial concentration of 5 × 10^4^ cells/mL in 96-well culture plates. After 24 h of incubation in a humidified incubator at 37 °C with 5% CO_2_, the cell viability of MDA-MB-231 cells was determined by co-culturing with different concentrations of Fe-CDs and FG (50, 100, 200, and 400 μg/mL) for 1, 3 and 5 days. Then, the cells were further incubated for 6 h and the cell culture medium was replaced with 100 μL of fresh media. To assess the cytotoxicity, each well containing 100 μL of the above medium was incubated for 4 h at 37 °C with 5% CO_2_ after adding 10 μL of MTT reagent. After incubation, 100 μL of lysis buffer (99.4% DMSO and 0.6% acetic acid) was added to the wells and incubated for another 15 min at room temperature. Finally, the absorbance of each group was measured at 570 nm using a microplate reader. Flow cytometry was used to detect cell apoptosis in the presence of different concentrations of Fe-CDs. The MDA-MB-231 cells were incubated with a diluted Fe-CDs solution (0, 6.25, 12.5, 25, 50, and 100 μg/mL). Then, the apoptosis level was determined using FITC Annexin V/PI apoptosis detection kit (C2015M, Beyotime, China). The flow cytometry data was analyzed using Flow Jo software (Flow Jo LLC, Ashland, OR, USA), where FITC(−)/PI(−) represented viable cells, FITC( +)/PI(−) represented early apoptotic cells, and FITC( +)/PI( +) represented late necrosis. The percentage of apoptosis was calculated by summing FITC( +)/PI(−) (early apoptosis) and FITC( +)/PI( +) (late apoptosis) cells. The above experiments were conducted three times to estimate errors.

### Intracellular localization assay

Intracellular location of Fe-CDs was determined by cellular iron accumulation assay using Prussian blue stain (G1426, Solarbio, China) according to the manufacturer’s instructions. The MDA-MB-231 cells and mouse mononuclear macrophage leukemia cells (RAW 264.7) that were incubated with a certain concentration of Fe-CDs were washed three times by double distilled water and fixed with paraformaldehyde for 15 min. Then, Prussian stain A and B were mixed 1:1, and added into the cells. The intracellular iron level was evaluated after 30 min visible of blue staining. After that, the samples were washed three times with ultrapure water, and the cells were stained for 1 min with Perls compound solution. The cells were observed under a light microscope, and the iron levels were determined based on the color intensities measured by Image J (ImageJ software, Bethesda, MD, USA).

### Quantitative real-time PCR (qPCR) assay

To mimic macrophages within the tumor cell microenvironment, RAW 264.7 cells were cultured with MDA-MB-231 cell medium with or without the addition of Fe-CDs. Total RNA was isolated from RAW 264.7 cells using TRIzol reagent (T9424, Sigma-Aldrich, USA) according to the manufacturer’s instructions. The gene expression levels were analyzed by real-time quantitative PCR (qRT-PCR). First-strand cDNA was synthesized from 2 μg total RNA in a 10 μL reaction mixture using a cDNA synthesis kit (RR036A, TAKARA, Japan) according to the manufacturer’s instructions. The as-prepared cDNA (2 μL) was used as a template for qRT-PCR. The qRT-PCR was performed using 2 × Real Star Fast SYBR qPCR Mix (SYBR Green with ROX; Enzynomics, Genster, China). The gene-specific primers used for qRT-PCR analysis were obtained from Shanghai Bioengineering Co., LTD. The IL-10 forward primer sequences were: 5′-CAGAGCCACATGCTCCTAGA-3′; the IL-10 reverse primer sequences were: 5′-TGTCCAGCTGGTCCTTTGTT-3′. The mouse α-actin forward primer sequences were: 5′-CTCCCTGGAGAAGAGCTATGA-3′; the mouse α-actin reverse primer sequences were: 5′-CCAAGAAGGAAGGCTGGAAA-3′). The mouse Gapdh forward primer sequences were: 5′-AACGACCCCTTCATTGAC-3′; the mouse Gapdh reverse primer sequences were: 5′-TCCACGACATACTCAGCAC-3′).

### Enzyme linked immunosorbent assay (ELISA)

The cell samples were collected from the cell supernatant, and diluted to a suitable concentration using the dilution buffer provided with the ELISA kit (PI536, Beyotime, China). The diluted samples were added to the wells of a microplate pre-coated with anti-IL-10 antibody, and the plate was incubated at room temperature or at 37 °C for a specified time in order to allow the IL-10 antigens to bind to the immobilized antibody. Then, the plate was incubated for 20 min to allow the substrate to react with the HRP conjugate and generate a color signal. Chromogenic TMB solution (100 μL/well) was added and incubated at room temperature in the dark for 20 min. The reaction was terminated by adding a stopping solution and then the absorbance of the wells was measured using a microplate reader. The concentration of IL-10 in each sample was calculated based on a standard curve generated using known concentrations of IL-10.

### Inhibition of epithelial-mesenchymal transition (EMT) experiment

To test the effect of Fe-CDs on migration of MDA-MB-231 cells, an in vitro scratch assay was performed. The cells in the logarithmic growth phase were selected and the single-cell suspensions were prepared. The MDA-MB-231 cells were further cultured in a 6-well plate until they reached 90% confluence. Then, a 200 μL pipette tip was used to create a scratch on the plate, after which the cells were cultured in the medium without serum. After 12 h, the width of the scratch was observed under a microscope, and the percentage of cell migration was calculated as follows:

$$\frac{{\left( {{\text{original scratch width}} - {\text{scratch width after migration}}} \right)}}{{\text{original scratch width}}}\, \times \,100\%$$

To further validate the cell migration assay, 12-well transwell chambers (3422, Corning Costar, USA) were used to perform transwell migration assays. Briefly, 1 × 10^4^ cells were plated in the upper transwell chamber and incubated with a certain concentration of 50 μg/mL Fe-CDs and CoCl_2_ in serum-free medium. Then, 0.5 mL complete medium was added into the lower chamber as chemoattractant. After 24 h of incubation, migrated cells on the membrane of the chambers were fixed with 4% paraformaldehyde at room temperature. After 15 min, cell staining with 0.1% crystal violet (C0121, Beyotime, China) was performed, followed by destaining with PBS solution. The number of migrating cells was counted under a microscope in five predetermined fields.

### Animal experiments

Thirty BALB/c mice aged 4–6 weeks were purchased from Beijing Huafukang Biotechnology Co., Ltd. During the experiments, the welfare of the experimental animals was fully ensured and the pain of the experimental animals was minimized as possible. After one week of normal rearing, 1.5 × 10^6^ mouse breast cancer cells (4T1) were injected subcutaneously into the right hind limb of each mouse. After 6 days of observation, the average tumor volume reached 100 mm^3^. The mice were then divided into two groups: the control group and the Fe-CDs administration group. The Fe-CDs drugs were injected intravenously every 3 d (2 mg/20 g mice body weight per day) until 21 d, with the control group being injected with the same volume of normal saline. The tumor volume and body weight of each mouse were recorded during therapy every 3 days.

### Immunofluorescence (IF) assay

Tumor tissues were collected from the anesthetized mice, and washed with cold PBS. The fixed tumor tissue was cut into 10 μm thickness by Leica cryostat (Leica) and attached to a gelatin-coated slide. The tissue sections and cultured cells were fixed in 4% polyformaldehyde (P1110, Solarbio, USA) for 15 min, and were then permeabilized with 0.3% Triton X-100 (P0096, Beyotime, USA) in PBS for 15 min, and incubated in 1% Bovine Serum Albumin (BSA) for 1 h at room temperature. Samples were then incubated at 4 °C overnight with primary antibodies diluted in 1% BSA. The primary antibodies used were as follows: IL-10 (1:100, ab133575, Abcam, U.K.). After washing with PBS, the samples were stained with the isotype-matched secondary antibodies for 2 h at room temperature away from light. An Alexa Fluor® 488-conjugated goat anti-rabbit IgG polyclonal (1:100, ab150077, Abcam, U.K.) was used as the secondary antibody. The fluorescence-labeled cells were analyzed using an Axiovert S100 fluorescence microscope equipped with an Axiocam digital camera.

### Hematoxylin–eosin (H&E) and immunohistochemistry (IHC) assay

Polyoxymethylene-fixed and paraffin-embedded sections were prepared from tumor and major organ tissues, and the embedded samples were sliced at 5 mm. Hematoxylin–eosin (H&E) was used for histological examination of the tumors and major organs, which were then observed under a bright field microscope. Immunohistochemistry (IHC) was used to stain tumor tissue, with the primary antibodies (Abcam, U.K.) used as follows: E-cadherin (1:500, ab40772), OCLN (1:200, ab216327), N-cadherin (1:500, ab51034), Vimentin (1:250, ab92547), Snail (1:5000, ab224731), Bcl-2 (1:250, ab32124), Caspase 3 (1:100, ab32351), IL-10 (1:200, ab217941), Arg-1 (1:100, ab183333). Then, the samples were stained with the isotype-matched secondary antibodies for 2 h at room temperature. The secondary antibodies was specific IHC polymer detection kit HRP/DAB (1:1000, ab209101, Abcam, U.K.). The stained tissues were observed under a bright field microscope.

### Western blot (WB) assay

For cell sample preparation, MDA-MB-231 cells were treated with a certain concentration of Fe-CDs, and the media from each group were collected to culture RAW 264.7 cells to simulate macrophages within the tumor microenvironment. The proteins from the corresponding groups of macrophages were extracted for analysis. Then, inhibitors of phosphatase and protease (P002, NCM, China) were added to RIPA buffer to lyse the cells and tissues, and total proteins from the cells were extracted from the supernatant. The protein concentrations of cell samples were measured with BCA Protein Assay Reagent (P0010S, Beyotime, China). After lysis, the proteins were separated by SDS-PAGE (150 V, 60 min) before being transferred to a PVDF membrane (GE Healthcare Life Sciences, Massachusetts, USA) with a Trans-Blot wet transfer (Bio-Rad, 400 mA, 25 min). The blots were then incubated with the primary antibody in fresh blocking buffer at 4 °C overnight. The primary antibody (Abcam, U.K.) used were listed as follows: Arg-1 (1:1000, ab183333), STAT3 (1:1000, ab68153), p-STAT3 (1:20,000, ab76315), P65 (1:1000, ab32536), p-P65 (1:1000, ab76302), JNK (1:20,000, ab179461), p-JNK (1:1000, ab124956), ERK (1:5000, ab184699), p-ERK (1:1000, ab201015), P38 (1:1000, ab31828), p-P38 (1:1000, ab4822), FN1 (1:1000, ab109365), N-cadherin (1:50,000, ab51034), Vemintin (1:1000, ab92547), α-SMA (1:100, ab5831), ZO-1 (1:1000, ab216880), OCLN (1:1000, ab216327), Snail (1:500, ab216347). After incubation with a HRP-conjugated secondary antibody for 2 h at room temperature, HRP signals were detected using a hypersensitive ECL Chemiluminescence Detection Kit (Proteintech Group). The results were analyzed using the densitometric analysis software Quantity One v4.6.2 for Windows (Bio‑Rad, CA, USA). The relative levels of target proteins were normalized by β-Actin (1:50000, AC026, ABclonal, China) and GAPDH(1:10,000, ab181602, Abcam, U.K.).

### Statistical analyses

Data are represented as mean values ± SEM. Statistical analysis was performed with the Prism 8.4/8.5 software (GraphPad Software). Normal distribution was tested by the Shapiro–Wilk method. If normality criteria was met, one-way ANOVA with post hoc multiple comparison test was performed; if normality criteria were not met, the Kruskal–Wallis non-parametric test was performed. (ns: not significant, *p < 0.05, **p < 0.01, ***p < 0.001, ****p < 0.0001).

## Results and discussion

### Synthesis and characterization of Fe-CDs

**Fig. 1:**
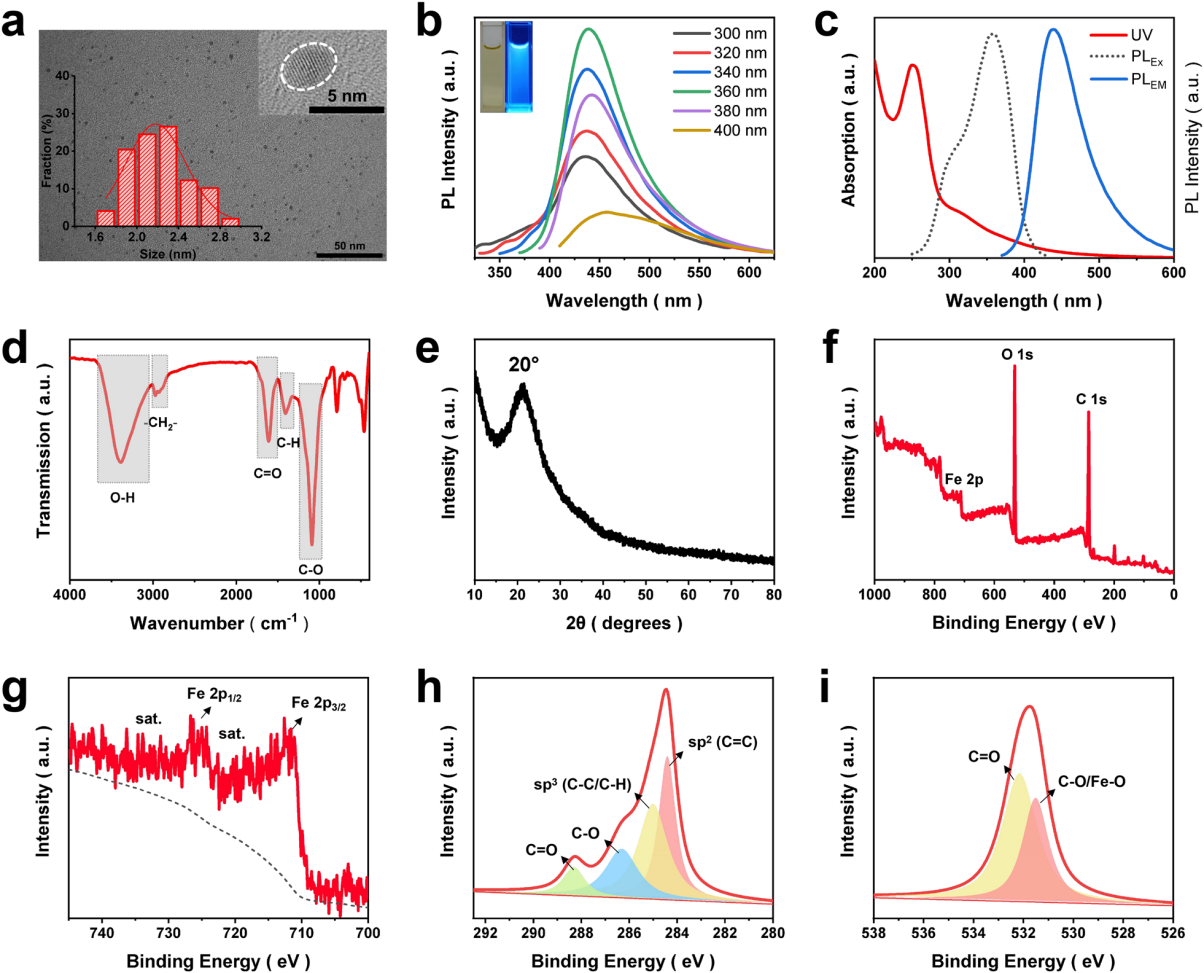
Characterization of Fe-CDs. a TEM images of Fe-CDs, insets show the size distribution histogram and the HR-TEM image. b PL spectra of Fe-CDs solution under different excitation wavelengths. c UV–Vis absorption spectra, the maximum excitation and emission PL spectra of Fe-CDs. d FT-IR spectrum of Fe-CDs. e XRD pattern of Fe-CDs. f XPS full spectra of Fe-CDs. g XPS deconvoluted Fe 2p spectra. h XPS deconvoluted C 1 s spectra, i XPS deconvoluted O 1 s spectra

In the present work, Fe-doped carbon dots (Fe-CDs) were derived from a Food and Drug Administration (FDA)-approved green iron supplement, i.e., ferrous gluconate, via an environmentally friendly one-step hydrothermal reaction. Our previous reports revealed that this kind of gluconic acid complex-based metal salt was suitable for the preparation of metal ions-doped CDs (MCDs) [34, 35]. Recently, MCDs had shown growing bioapplication prospects in the fields of diagnosis and therapy, due to the synergistic features of the nano-size effect, biocompatibility of CDs and specific functionality of metal ions [36]. Iron metabolism in the tumor-related cells was closely related to the occurrence and development of cancer, which inspired plenty of works on Fe-based anti-tumor nanomaterials, bringing cancer therapy into an iron age [37–39]. In this regard, it was speculated that the as-synthesized Fe-CDs might have potential as a novel iron agent for tumor treatment. First, the morphology and structure of Fe-CDs were investigated, as shown in Fig. 1a, the transmission electron microscopy (TEM) image showed that Fe-CDs appeared as uniform nanoparticle with an average diameter of 2.2 nm, such a small nanosize would facilitate the rapid entry of Fe-CDs into cells. The high-resolution TEM image revealed lattice fringes with 0.21 nm spacing, which was corresponding to the (100) crystal plane of graphite, indicating the graphitized carbon core within Fe-CDs [40] The photoluminescence (PL) spectrum of Fe-CDs exhibited an typical excitation wavelength-dependent blue fluorescence property (Fig. 1b) [41], and the maximum emission wavelength was 440 nm when excited under 358 nm light (Fig. 1c). There were two peaks observed in the UV–Vis absorption spectra of Fe-CDs that located at 252 and 316 nm, which could be attributed to the π → π* transition of C = C bond and n → π* transition of C = O bond, respectively [42]. Fourier transform infrared spectroscopy (FTIR) was adopted to study the functional groups of Fe-CDs, the stretching vibrations of O–H at 3386 cm^−1^, C = O at 1610 cm^−1^ and C-O at 1089 cm^−1^ (Fig. 1d), demonstrating the abundant oxygen-containing groups on the surface of Fe-CDs that endow it with good water dispersibility.

Then, the existing states of Fe within Fe-CDs was further determined. X-ray diffraction analysis of Fe-CDs displayed a broad peak at around 20°, corresponding to amorphous carbon (Fig. 1e) [43]. There were no characteristic peaks of iron oxide or elemental iron, which might be caused by the low iron content, or possibly due to the fact that Fe dopants in Fe-CDs were ionic form. This was possibly because the hydrothermal conditions were relatively mild and the insoluble oxide from the as-prepared products were also subjected to post-treatment by filtration. The existence of Fe in Fe-CDs was confirmed by energy dispersive spectrometer (EDS), which determined the element weight percentage was 72.1%, 18.2%, and 9.7% for C, O, and Fe, respectively (Additional file 1: Fig. S1). Besides, the Fe ion content was also accurately analyzed as 6.46 wt% by inductive coupled plasma emission spectrometer (ICP) (Additional file 1: Fig. S2), similar to the EDS result. In order to further understand the elemental composition of Fe-CDs and doping forms of Fe, X-ray photoelectron spectroscopy was conducted, which confirmed the existance of C, O, and Fe in Fe-CDs with the atomic percentage (At.%) of 64.9%, 28.9%, and 6.2%, respectively (Fig. 1f). The deconvoluted Fe 2p spectra of Fe-CDs possessed the signals of Fe 2p_3/2_ and Fe 2p_1/2_ electronic configurations at the binding energy of 711.6 and 724.9 eV, respectively, along with the adjacent satellite peaks (Fig. 1g). These were associated with the presence of ionic Fe^3+^ according to previous reports [44], which further confirmed the XRD analysis. The C 1 s spectra was divided into four types of carbon, i.e., sp^2^ C (C = C), sp^3^ C (C–C), C-O, and C = O at 284.4, 285.1, 286.3, and 288.4 eV, respectively (Fig. 1h), which was consistent with the FTIR results. The deconvoluted peaks in the O 1 s spectra at 532.2 and 531.5 eV could be ascribed to C = O and C-O/Fe–O bonds, respectively (Fig. 1i). Moreover, the chromatic reaction of Fe^3+^ and salicylic acid (SA) was introduced to determine the iron-doped state in Fe-CDs (Additional file 1: Fig. S3). It could be clearly seen that with the increase of the concentration of Fe-CDs added in SA solution, the purple red complex color deepened. However, there appeared no color change after the mixing of divalent iron salt FeSO_4_ and SA, indicating the Fe^3+^ doping status of Fe-CDs. In summary, a type of Fe ions-doped fluorescent CDs was successfully synthesized, in which Fe ions could be bound to oxygen-containing functional groups on the surface of CDs through electrostatic interactions or ionic bonds. It would be of great interest to investigate the role of this novel nano-iron agent in the regulation of TME and the application in anti-tumor.

### In vivo evaluation of anti-tumor effects of Fe-CDs

**Fig. 2:**
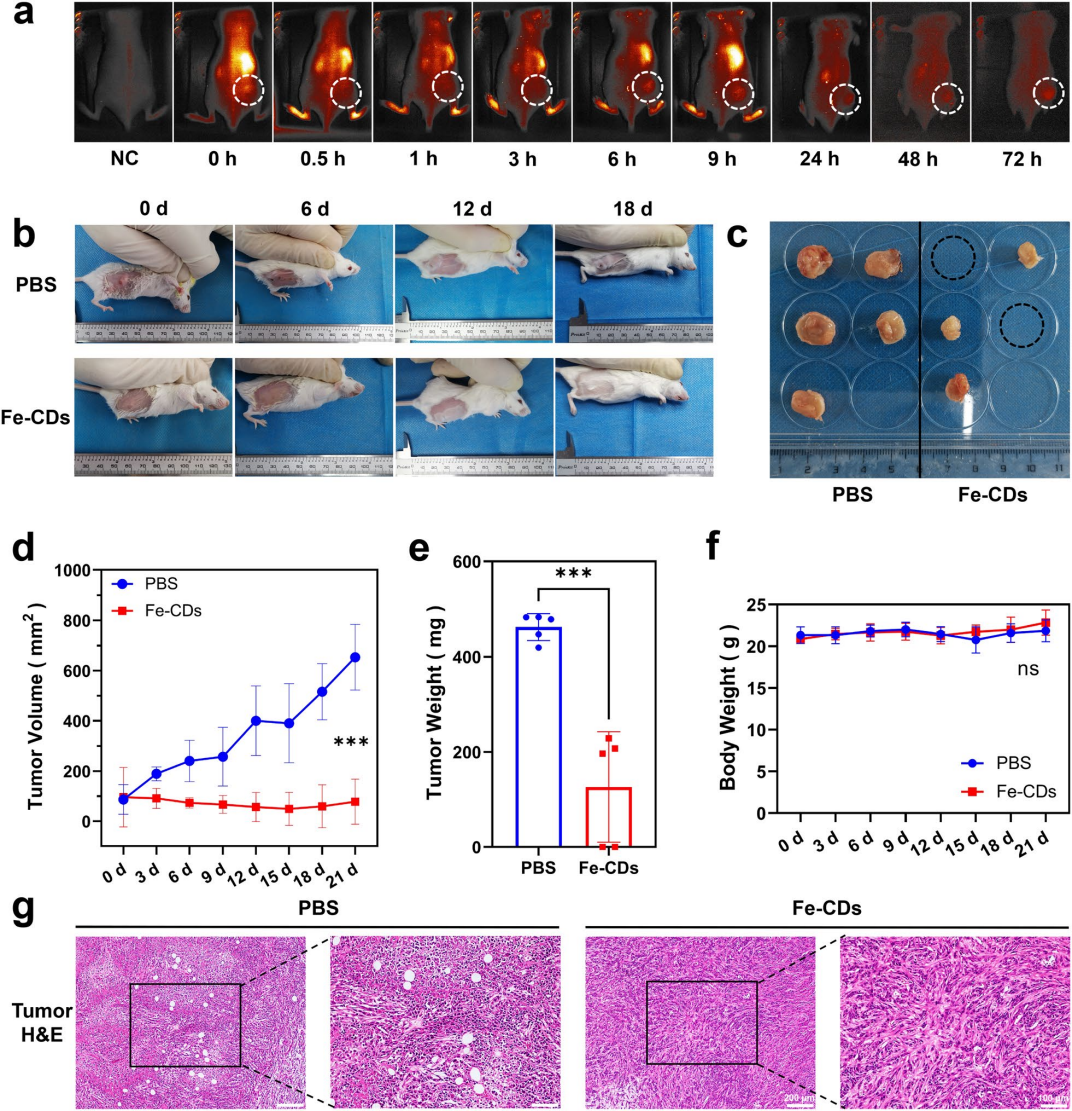
The in vivo evaluation of anti-tumor activity of Fe-CDs. a Distribution of Fe-CDs in tumor-bearing mice using an in vivo imaging system. b Photographs of tumor-bearing mice at different treatment times of PBS and Fe-CDs. c Photographs of tumors collected from different groups of mice after 21 d treatment of PBS (left) and Fe-CDs (right). d Mean tumor volume versus time curve of PBS and Fe-CDs treatment groups. e Mean weight of tumors of PBS and Fe-CDs treatment groups at 21 d. f Mean body weight of normal mice after intravenous administration of PBS and Fe-CDs for different days. g Histological analysis of cancerous tissues by H&E staining after treatments with PBS and Fe-CDs. (significance analysis was performed using one-way ANOVA method. ns: not significant, ***p < 0.001, n = 6)

An orthotopic tumor model was constructed by subcutaneous injection of 4T1 cells (mouse breast cancer cells) in the back of Balb/C mice, which was a classic tumor model suitable for preliminary evaluation of the Fe-CDs anti-tumor effect. First of all, the in vivo metabolic kinetics of Fe-CDs was studied by intravenous injection of Fe-CDs chemically coupled with Cyanine 5.5 (Cy5.5), which could achieve near-infrared intravital imaging with low interference of tissue autofluorescence. As shown in Fig. 2a, the fluorescence signal was mainly concentrated in the liver area of mouse during the first 3 h, consistent with the previous reports that CDs being metabolized by liver and kidney [45]. Then, Fe-CDs gradually accumulated to the tumor site from 6 h after injection until 72 h (white dashed area), which could be due to the enhanced permeability and retention effect (EPR) effect of nano-sized Fe-CDs, or the increased permeability of tumor blood vessels leading to the local uptake of Fe-CDs [46].

The circulating administration of Fe-CDs (2 mg/20 g body weight per day, injected once every 3 d) through tail vein was started when the subcutaneous tumor reached about 100 mm^3^, and the control group was an equal volume of PBS buffer solution injected intravenously (Fig. 2b). As we expected, the diameter of the resected tumor had grown to 12.56 ± 1.97 mm in the control group, while the tumor size of Fe-CDs group decreased to 6.74 ± 1.48 mm. Surprisingly, the tumors had completely disappeared in two groups of tumor-bearing mice (Fig. 2c), demonstrating the superior ability of Fe-CDs to resist tumors. The mean tumor volume gradually decreased and eventually shrank to about half of the initial value when Fe-CDs were administered every 3 days (Fig. 2d). However, the tumor volume in the PBS-injected group increased by more than five times. Also, the average tumor weight of the PBS group was about 3.7 times that of the Fe-CDs treatment group, indicating that Fe-CDs significantly inhibited the in-vivo proliferation of solid tumors (Fig. 2e).

**Fig. 3:**
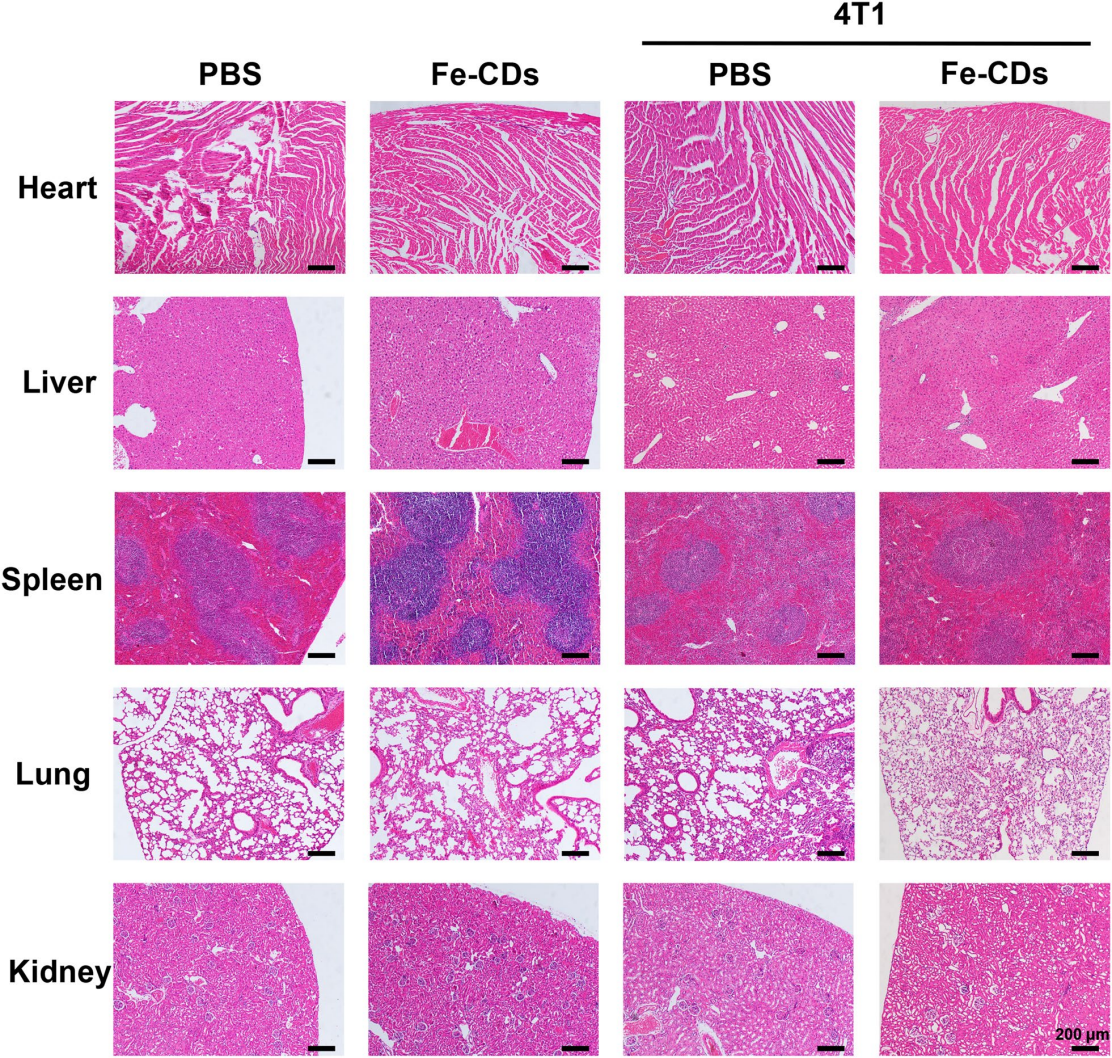
In vivo toxicology evaluation of Fe-CDs. Histological analysis of essential organs of normal mouse (left) and tumor-bearing mouse (right) by H&E staining after treatments with PBS and Fe-CDs

Moreover, systemic circulatory dosing of Fe-CDs had no significant influences on the mice weight, which demonstrated the extremely low toxicity of Fe-CDs (Fig. 2f). The hematoxylin–eosin staining (H&E) of tumor tissue illustrated that there were obvious necrotic areas and substantially remission of immune environment within the tumor area in Fe-CDs group compared with the PBS group, confirming that Fe-CDs could selectively induce tumor necrosis and ablation (Fig. 2g). It was worth mentioning that such an anti-tumor effect could be achieved by intravenous administration of Fe-CDs, without integrating other chemotherapy or phototherapy moieties, and targeting molecules. In order to further verify the biocompatibility of Fe-CDs to normal tissues, the vital organs (heart, liver, spleen, lung, kidney) of mice were dissected out for H&E staining, and the sections displayed no obvious changes of cell states and inflammatory infiltration in both the control group and Fe-CDs group, which also proved the excellent biocompatibility of Fe-CDs (Fig. 3). Therefore, a type of biocompatible Fe-CDs with exceptional anti-tumor activity was simply synthesized, which showed great potential as a target-free, low-toxicity, and effective anti-tumor nanodrug. It was necessary to explore and clarify the mechanism for the efficient tumor treatment effect of Fe-CDs, so as to provide guidance and reference for the preparation of Fe-based anti-tumor nanomaterials.

### Cytotoxicity and ability of Fe-CDs to induce tumor cell apoptosis

**Fig. 4:**
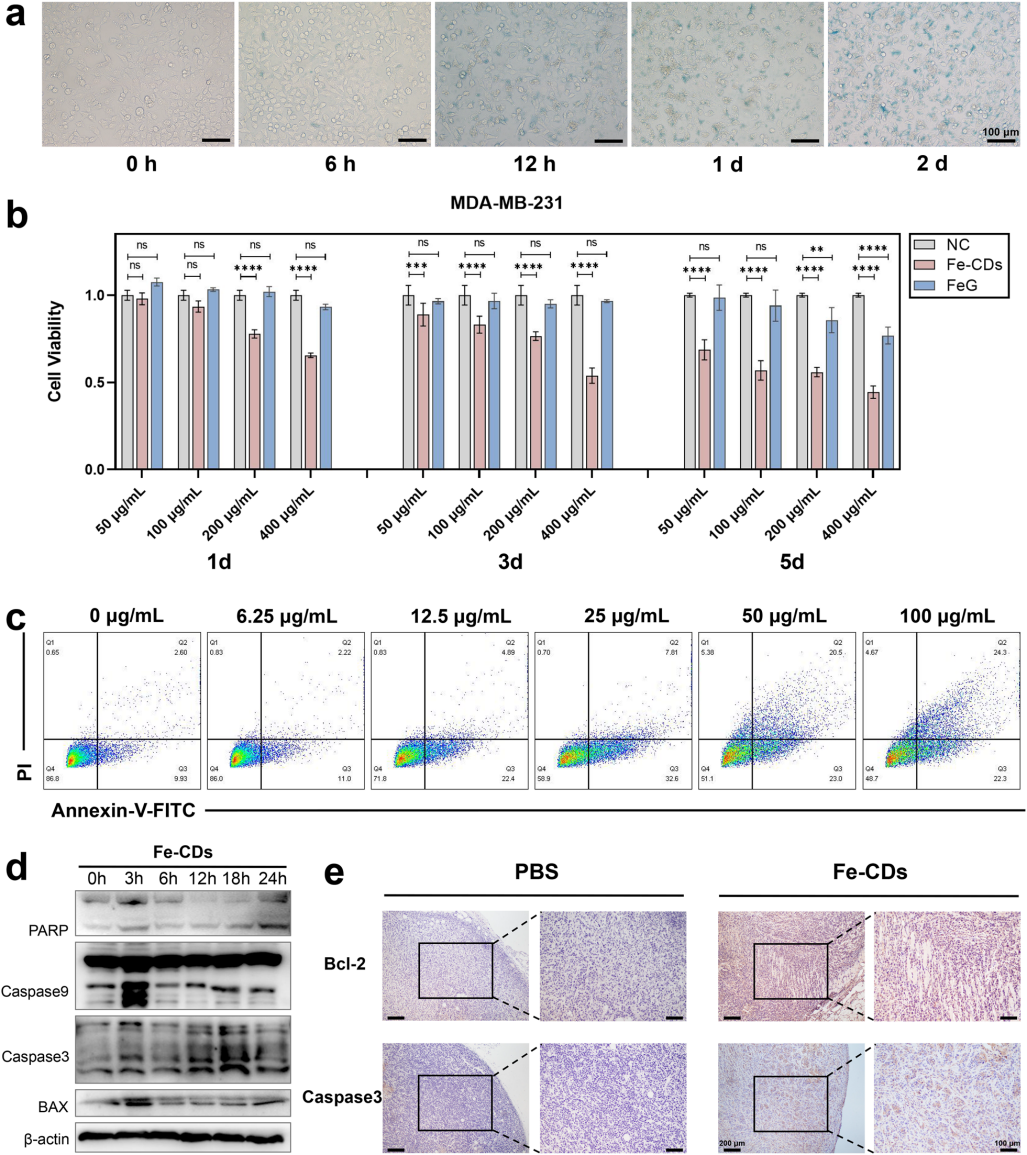
The effect of Fe-CDs on tumor cell viability and apoptosis. a Prussian blue staining of Fe in tumor cells incubated with Fe-CDs for different time. b Cell viability of tumor cells incubated with Fe-CDs and FeG by MTT assay. c Cell apoptosis of tumor cells incubated with different concentration of Fe-CDs by flow cytometry. d Western blot of expression levels of apoptosis-related proteins in tumor cells treated with Fe-CDs at different incubation time. e Immunohistochemical staining of apoptosis marker in cancerous tissues treated with PBS (left) and Fe-CDs (right). (ns: not significant, **P < 0.01, ***P < 0.001, ****P < 0.0001, n = 6)

Before conducting in vitro anti-tumor related experiments, the cellular localization and cytotoxicity needed to be assessed. Prussian blue staining was used to locate Fe-CDs in human triple-negative breast cancer cells (MDA-MB-231) by labeling ferric irons (Fig. 4a). After 6 h of co-culture with Fe-CDs, there were visible blue markers around partial cells, confirming initial cellular uptake of Fe-CDs. In addition, some cells showed morphological changes, nuclear pyknosis, and destruction of cell membrane after 12 h. With the extension of incubation time till 48 h, the content of Fe-positive labeled cells and necrotic cells gradually increased, which suggested that Fe-CDs could be enriched in MDA-MB-231 cells and induce cell death. Moreover, TEM images of tumor cell sections that co-cultured with Fe-CDs also clearly showed the presence of Fe-CDs in the state of aggregated particles within the cytoplasm in 2 h (Additional file 1: Fig. S4), indicating that they could enter the tumor cells quickly.

Then, the cytotoxicity experiment of Fe-CDs was performed by MTT assay, and the raw material ferrous gluconate was used as a control to define the specific role of preparing Fe-CDs. It could be seen that Fe-CDs significantly led to MDA-MB-231 cell death with a time- and concentration-dependent manner (Fig. 4b), when the culture condition was 400 μg/mL of Fe-CDs for 3 d and 100 μg/mL for 5 d, the cell viability reached (53.8 ± 0.031)% and (55.9 ± 0.019)%, respectively, in other words, nearly half of the cancer cells died under Fe-CDs treatment. In contrast, the raw material compound, i.e., ferrous gluconate (FeG), had almost no cytotoxicity to tumor cells, similar to the control group. It was speculated that due to the improved water solubility and cell entry mode of iron drugs after nanometerization, which made it easier to be taken up by cells, and the exposure of iron on the surface of carbon dots was also conducive to its effect. It was worth mentioning that Fe-CDs could selectively kill tumor cells without showing obvious toxicity to normal cells, such as human dental pulp stem cells (hDPSCs) and human umbilical vein cell fusion cells (EA.hy926), with cell viability above 80% at all concentrations and time gradients (Additional file 1: Fig. S5–S6), indicating the the potential of Fe-CDs as biocompatible antitumor drugs.

In order to confirm how Fe-CDs caused tumor cell death, the effects of different inhibitors on cell activity were investigated. The cell ferroptosis inhibitor (Fer) and necrosis inhibitor (Nec) showed restraining effects on the decrease of cell viability caused by Fe-CDs, especially cell apoptosis inhibitor (ZVAD) significantly reversed the Fe-CDs induced cell death (Additional file 1: Fig. S7), indicating that Fe-CDs kill tumor cells mainly through apoptosis pathway. Generally, ferroptosis was mainly caused by lipid peroxidation catalyzed by divalent Fe ions [47]. Although the as-prepared Fe-CDs was a type of Fe-doped nanomaterials, the Fe within Fe-CDs existed mainly in Fe^3+^ form, so the tumor cell death caused by Fe-CDs was not mainly through ferroptosis way. In addition, the doping amount of Fe in Fe-CDs was relatively low, it was believed that the nano-structures and surface groups of CDs also played an important synergistic role in the improved anti-tumor function and biocompatibility of Fe-CDs. Further, Annexin-V and PI double staining was introduced to detect apoptosis by flow cytometer, which was shown in Fig. 4c. When the concentration of Fe-CDs was low (< 25 μg/mL), early apoptosis of MDA-MB-231 cells mainly occurred (Q3) and the apoptosis rate elevated with the increase of Fe-CDs concentration. Then late apoptosis started to rise (Q2) and became dominant as the concentration of Fe-CDs further increased to 100 μg/mL, representing that the progression of apoptosis was exacerbated by high Fe-CDs concentration. It was further verified by Western Blot that Fe-CDs induce tumor cell apoptosis rather than other death pathways. The addition of Fe-CDs activated the expression of apoptosis-related protein including PARP, Caspase 3, Caspase 9, and BAX with a time-dependent characteristic (Fig. 4d, Additional file 1: Fig. S8). Moreover, immunohistochemical staining of apoptosis-related markers was performed in tumor tissue sections, namely B-cell CLL/lymphoma 2 (Bcl-2) and Caspase 3, to further determine the ability of Fe-CDs in inducing tumor apoptosis. The up-regulation of apoptosis level in Fe-CDs group could be clearly observed as compared to the negative control (NC) group (Fig. 4e). It is worth noting that G-CDs without Fe doping did not have tumor cytotoxicity and failed to induce apoptosis of tumor cells (Additional file 1: Fig. S9–S11), indicating that the ability of Fe-CDs to cause tumor cell death is inseparable from Fe dopants. The above results demonstrated that Fe-CDs possessed the function of inducing tumor cell apoptosis, which contributed to its excellent anti-tumor effect in vivo.

### Ability of Fe-CDs to activate the immunity of antitumoral macrophages

**Fig. 5:**
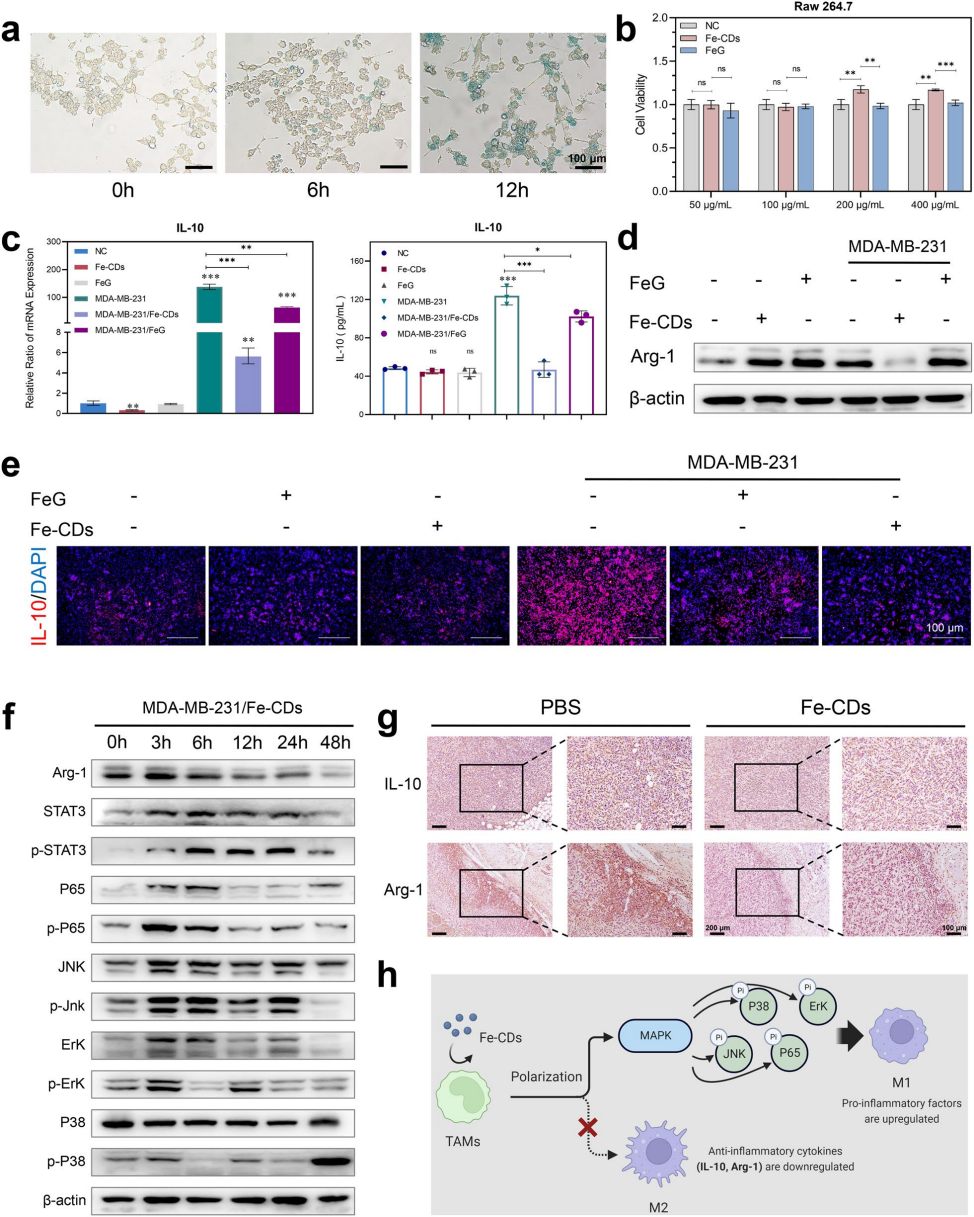
The ability of Fe-CDs to modulate macrophage polarization. **a** Prussian blue staining of Fe in macrophages incubated with Fe-CDs for different time. **b** Cell viability of macrophages incubated with Fe-CDs and FeG by MTT assay. **c** qPCR assay (left) and ELISA assay (right) of IL-10 expression level in macrophages with (right) and without (left) tumor cell medium, in the presence of Fe-CDs or FeG. **d** Western blot of Arg-1 expression levels in macrophages incubated with (right) or without (left) tumor cell medium, in the presence (+) or absence (−) of Fe-CDs and FeG. **e** Immunofluorescence images of IL-10 in macrophages with or without tumor cell medium, in the presence or absence of Fe-CDs and FeG. **f** Western blot of expression levels of M2-type macrophage-related proteins in macrophages treated with tumor cell medium in the presence of Fe-CDs at different incubation time. **g** Immunohistochemical staining of M2-type macrophage marker in cancerous tissues treated with PBS (left) and Fe-CDs (right). **h** Schematic representation of the ability of Fe-CDs in modulating macrophage polarization (Created with BioRender.com). (ns: not significant, *P < 0.01, **P < 0.001, ***P < 0.0001, n = 6)

Immunotherapy had become the most promising anti-cancer therapies in the clinic because it utilized the mobilization of autoimmune systems to achieve tumor eradication with extremely low side effects and recurrence rates. In tumor microenvrionment (TME), Fe was preferentially taken up by the surrounding tumor-associated macrophages (TAMs), forming iron-carrying macrophages [48, 49]. Previous reports had found that an FDA-approved iron agent, i.e., ferumoxytol (nano-iron oxide), could inhibit tumor cell proliferation by modulating macrophages functions [39, 50]. Regarding this, the effects of Fe-CDs on TAMs immunity were further explored. Similarly, the localization of Fe-CDs in mouse mononuclear macrophage leukemia cells (RAW 264.7) was also achieved by Prussian blue staining, showing that Fe-CDs began to enter cells within 1 day, and the degree of entry further increased at 3 days (Fig. 5a), which confirmed that Fe-CDs could be enriched in macrophages. Unexpectedly, both Fe-CDs and FeG showed no significant cytotoxicity to macrophages in a certain concentration range (Fig. 5b), that is, Fe-CDs could selectively kill tumor cells without side effects on other kinds of cells, and this performance was extremely important for the practical application of anti-tumor nanomedicines.

Then, quantitative real-time polymerase chain reaction (qPCR) and enzyme linked immunosorbent assay (ELISA) were implemented to analyze the expression level of Interleukin-10 (IL-10) in macrophages under the presence of Fe-CDs. The cytokine IL-10 was found to be abundantly secreted by activated macrophages in TME, making the phenotype of TAMs tend to be anti-inflammatory M2, which could inhibit the immune response and specific tumor killing [51, 52]. Here, it could be seen that Fe-CDs had no significant effect on IL-10 generation in macrophages under normal conditions (Fig. 5c). However, when macrophages cultured in tumor cells culture medium were used to mimic TAMs, the mRNA transcription and protein expression of IL-10 were obviously down-regulated by the addition of Fe-CDs. In other words, the differentiation of pro-inflammatory M1 type TAMs was promoted through inhibiting IL-10 secretion by Fe-CDs, which could awaken the immune function of antitumoral macrophages to selectively kill tumor cells. Noteworthy, the primitive drug FeG showed a relatively weaker activity than Fe-CDs due to the poor solubility and uptake rates. Also, Fe-free G-CDs did not have the capability to reverse the expression of IL-10 in TAMs (Additional file: Figure S12), indicating the importance of both Fe doping and CDs nanocarrier for Fe-CDs to activate macrophages immunity. The results of immunofluorescence staining further proved the conclusion that the tumor cell culture significantly stimulate macrophages to secrete IL-10, which could be effectively restrained by Fe-CDs (Fig. 5d). Moreover, another two typical soluble mediator secreted by M2 macrophages, Arginase-1 (Arg-1) and phosphorylation of P38 (p-P38), could serve as the specific markers for M2 polarization [52, 53]. It could be clearly seen that the presence of Fe-CDs significantly reduced the expression of Arg-1 and p-P38 in macrophages cultured in tumor cell medium, whereas FeG had almost no effect on Arg-1 and p-P38 levels (Fig. 5e, Additional file 1: Fig. S13–S14). In addition, compared with untreated TAMs (Additional file 1: Fig. S15–S16), Fe-CDs also markedly inhibited the phosphorylation levels of signal transducer and activator of transcription 3 (STAT3), c-Jun N-terminal kinase (p-JNK), extracellular regulated protein kinases (ERK), and P65 with a time-dependent tendency (Fig. 5f, Additional file 1: Fig. S17), which both belonged to the Mitogen-activated protein kinases (MAPK) signaling pathway. According to the previous reports, the activation of MAPK was essential for the antitumoral macrophages [54]. Moreover, the in vivo histochemical staining results of IL-10 and Arg-1 in tumor tissues further demonstrated that Fe-CDs stimulated antitumoral macrophages by inhibiting the expression of IL-10/Arg-1 (Fig. 5g). Based on the above results, it was concluded that the as-prepared Fe-CDs could modulate tumor immunity by the macrophage IL-10/Arg-1 routes, and promoted the transformation of macrophages to the tumor-specific killer M1 phenotype through the MAPK pathway (Fig. 5h), especially the regulating ability was stronger than that of FeG due to the improved internalization efficiency after nanoization.

### Ability of Fe-CDs to inhibit epithelial-mesenchymal transition (EMT) process

**Fig. 6:**
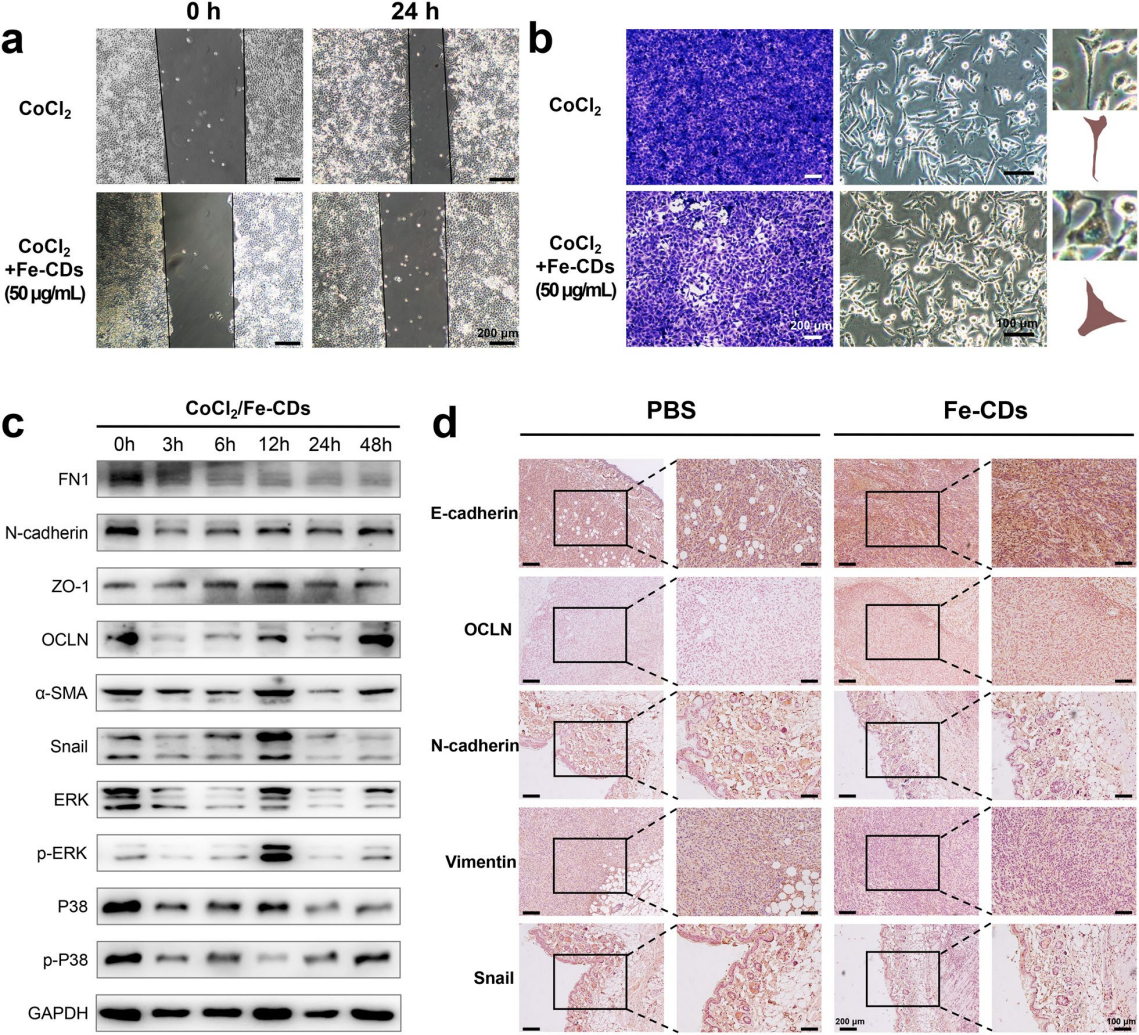
The effect of Fe-CDs on the EMT of tumor cells. **a** Scratch test of the effect of CoCl_2_ and Fe-CDs on the migration ability of tumor cells. **b** Transwell assay (left) and cell morphology images (right) of the effect of CoCl_2_ and Fe-CDs on the migration ability of tumor cells. **c** Western blot of expression levels of EMT-related proteins in tumor cells treated with CoCl_2_ and Fe-CDs at different incubation time. **d** Immunohistochemical staining of EMT marker in cancerous tissues treated with PBS (left) and Fe-CDs (right)

The metastasis of tumor cells, which led to the recurrence and spread of malignant tumors, had been considered as the most important factor in the incurability of cancer [55]. In recent years, it was found that the programmed activation of epithelial-mesenchymal transition (EMT) could make tumor cells lose the tight junction and adhesion between cells, and then switched to the morphology of mesenchymal cells with strong migratory ability [56]. Surprisingly, the as-synthesized Fe-CDs possessed the unique ability to inhibit tumor cell migration and EMT. First, the scratch assay images of MDA-MB-231 cells cultivated with Fe-CDs exhibited a significant reduction of proliferation rate compared to the control group that incubated in phosphate buffered saline (PBS) (Additional file 1: Fig. S18a). The migration rate of the control group in scratch assay was 57%, while the migration rate of cells treated with Fe-CDs was reduced to almost 6% (Additional file 1: Fig. S19a). Noteworthy, G-CDs without Fe doping did not have the ability to inhibit tumor cell migration (Additional file 1: Fig. S20), that is, the function of Fe-CDs to prevent EMT was related to the doped Fe, which might be due to the charge repulsion of Fe ions or the influence of EMT-related signaling pathways. Then, the morphology of tumor cells observed under microscope changed from cobblestone-like to spindle-like shape, indicating the loss of cell polarity and increased migratory capacity (Additional file 1: Fig. S18b). However, the existence of Fe-CDs (50 μg/mL) could partially reverse the cytoskeletal changes to a certain extent, representing its potential in suppressing EMT of tumor cells. In addition, cobalt chloride (CoCl_2_) was introduced to further explore the function of Fe-CDs in suppressing EMT, as an appropriate amount of CoCl_2_ had been reported to induce mesenchymal cell transformation [57]. Similar to the above experiments, it could be seen that although CoCl_2_ accelerated the cell migration process, the addition of Fe-CDs could still effectively prevent tumor cells from infiltrating into the scratch (Fig. 6a). The migration rate of cells treated with CoCl_2_ was 50%, while the migration rate of tumor cells co-treated with CoCl_2_ and Fe-CDs was reduced to 8% (Additional file 1: Fig. S19b). Also, the exacerbated process of cell morphological transformation was alleviated by Fe-CDs, that is, in the presence of Fe-CDs, the tumor cells could be converted from aggressive spindle shape to conservative round shape (Fig. 6b). These results were further confirmed by the transwell migration experiments shown on the left side of Fig. 6b, which clearly depicted that the number of chemotactic tumor cells in both the control and CoCl_2_ groups was significantly higher when Fe-CDs was absent.

Furthermore, several EMT-related markers were performed by WB analysis, including Fibronectin (FN1), N-cadherin, Vemintin, α-Smooth muscle actin (α-SMA), Snail, which had been reported to induce EMT, and also Zonula occludens-1 (ZO-1), Occludin (OCLN), which could inhibit EMT (Additional file 1: Fig. S21) [58]. It could be seen that Fe-CDs treatment significantly reduced the expression of EMT positive-associated proteins and showed an increase in the expression level of EMT inhibitory proteins with a concentration-dependent manner (Additional file 1: Fig. S22). The obvious up-regulation of p-P38 and p-ERK with the addition of Fe-CDs also suggested that EMT process might be inhibited through related pathways. Similarly, when CoCl_2_ was introduced, the above proteins were also subjected to WB test, which confirmed that they could accelerate the EMT process of tumor cells over time (Additional file 1: Fig. S23), whereas Fe-CDs effectively counteracted the progress of EMT (Fig. 6c, Additional file 1: Fig. S24–S25). Furthermore, the function of Fe-CDs to inhibit EMT was also verified in vivo by staining with the antibodies of E-cadherin, OCLN, N-cadherin, Vimentin and Snail, similar to the WB assay in cells (Fig. 6d). The above results demonstrated that Fe-CDs might have the potential to suppress cancer migration by blocking the EMT process of tumor cells. Since the as-synthesized Fe-CDs had been proved to be equipped with three-step cancer therapy functions, i.e., inducing tumor cell apoptosis, modulating macrophage immunity, and inhibiting EMT, leading to the superior anti-tumor effect of Fe-CDs in vivo without integrating other moieties and external stimuli, it was believed that Fe-CDs could serve as a promising alternative to tumor immunotherapy agents and anti-tumor nanoplatform.

## Conclusions

In summary, a novel type of “green” Fe-CDs with multi-level anti-tumor ability was synthesized. In vitro experiments surprisingly found that Fe-CDs could simultaneously induce tumor cell apoptosis, activate antitumoral macrophages immunity, and inhibit the EMT process. Based on the multiple function and excellent biocompatibility, intravenous administration of Fe-CDs could achieve significant selective inhibition and killing of tumors without the conjugation of external stimuli or targeting moieties. In particular, continuous treatment of Fe-CDs could prevented tumor growth without significant systemic toxicity and body weight fluctuations. Taken together, the as-synthesized Fe-CDs in this study revealed great prospect as high-efficiency anti-tumor drugs with low side effects, and might show potential applications when serving as a new nanoplatform to further combined with other drugs and therapies.

### Supplementary Information

**Additional file 1:** Additional figures S1–S25.

## Data Availability

All data generated or analyzed during this study are included in this article and its additional file.

## Abbreviations

CDs: Carbon dots
Fe-CDs: Fe ions-doped carbon dots
MCDs: Metal ions-doped CDs
EMT: Epithelial-mesenchymal transition
TME: Tumor microenvironment
TAMs: Tumor-associated macrophages
SNPs: Single-component nanoparticles
FG: Ferrous gluconate dihydrate
Cy5.5: Sulfo-Cyanine 5.5
PBS: Phosphate buffered saline
DMEM: Dulbecco’s modified Eagle’s medium
RPMI: Roswell Park Memorial Institute medium
FBS: Foetal bovine serum
BSA: Bovine serum albumin
TEM: Transmission electron microscope
EDS: Energy dispersive X-ray spectrometer
PL: Photoluminescence
UV–vis: Ultraviolet–visible
FTIR: Fourier transformed infrared spectra
XRD: X-ray diffraction
XPS: X-ray photoelectron spectroscopy
ICP: Inductively coupled plasma spectrometer
hDPSCs: Human dental pulp stem cells
DMSO: Dimethyl sulfoxide
qRT-PCR: Real-time quantitative polymerase chain reaction
ELISA: Enzyme linked immunosorbent assay
IF: Immunofluorescence
H&E: Hematoxylin–eosin
IHC: Immunohistochemistry
WB: Western blot

## References

[1] Xu J, Shamul JG, Wang H, Lin J, Agarwal P, Sun M, Lu X, Tkaczuk KHR, He X (2020). Targeted heating of mitochondria greatly augments nanoparticle-mediated cancer chemotherapy. Adv Healthc Mater.

[2] Li H, Wang M, Huang B, Zhu SW, Zhou JJ, Chen DR, Cui R, Zhang M, Sun ZJ (2021). Theranostic near-infrared-IIb emitting nanoprobes for promoting immunogenic radiotherapy and abscopal effects against cancer metastasis. Nat Commun.

[3] He S, Jiang Y, Li J, Pu K (2020). Semiconducting polycomplex nanoparticles for photothermal ferrotherapy of cancer. Angew Chem Int Ed Engl.

[4] Ding D, Zhong H, Liang R, Lan T, Zhu X, Huang S, Wang Y, Shao J, Shuai X, Wei B (2021). Multifunctional nanodrug mediates synergistic photodynamic therapy and MDSCs-targeting immunotherapy of colon cancer. Adv Sci (Weinh).

[5] Hou L, Chen D, Wang R, Wang R, Zhang H, Zhang Z, Nie Z, Lu S (2021). Transformable honeycomb-like nanoassemblies of carbon dots for regulated multisite delivery and enhanced antitumor chemoimmunotherapy. Angew Chem Int Ed Engl.

[6] Agarwal Y, Milling LE, Chang JYH, Santollani L, Sheen A, Lutz EA, Tabet A, Stinson J, Ni K, Rodrigues KA (2022). Intratumourally injected alum-tethered cytokines elicit potent and safer local and systemic anticancer immunity. Nat Biomed Eng.

[7] Ma S, Sun B, Duan S, Han J, Barr T, Zhang J, Bissonnette MB, Kortylewski M, He C, Chen J (2023). YTHDF2 orchestrates tumor-associated macrophage reprogramming and controls antitumor immunity through CD8(+) T cells. Nat Immunol.

[8] Mantovani A, Allavena P, Marchesi F, Garlanda C (2022). Macrophages as tools and targets in cancer therapy. Nat Rev Drug Discov.

[9] Lu W, Kang Y (2019). Epithelial-mesenchymal plasticity in cancer progression and metastasis. Dev Cell.

[10] Ma X, Yao M, Shi J, Li X, Gao Y, Luo Q, Hou R, Liang X, Wang F (2020). High intensity focused ultrasound-responsive and ultrastable cerasomal perfluorocarbon nanodroplets for alleviating tumor multidrug resistance and epithelial-mesenchymal transition. ACS Nano.

[11] Saeed M, Chen F, Ye J, Shi Y, Lammers T, De Geest BG, Xu ZP, Yu H (2021). From design to clinic: engineered nanobiomaterials for immune normalization therapy of cancer. Adv Mater.

[12] Musetti S, Huang L (2018). Nanoparticle-mediated remodeling of the tumor microenvironment to enhance immunotherapy. ACS Nano.

[13] Cai Y, Chen X, Si J, Mou X, Dong X (2021). All-in-one nanomedicine: multifunctional single-component nanoparticles for cancer theranostics. Small.

[14] Monro S, Colón KL, Yin H, Roque J, Konda P, Gujar S, Thummel RP, Lilge L, Cameron CG, McFarland SA (2018). Transition metal complexes and photodynamic therapy from a tumor-centered approach: challenges, opportunities, and highlights from the development of TLD1433. Chem Rev.

[15] Xue C, Li M, Liu C, Li Y, Fei Y, Hu Y, Cai K, Zhao Y, Luo Z (2021). NIR-actuated remote activation of ferroptosis in target tumor cells through a photothermally responsive iron-chelated biopolymer nanoplatform. Angew Chem Int Ed.

[16] Chen T, Zeng W, Liu Y, Yu M, Huang C, Shi Z, Lin C, Tang J, Mei L, Wu M (2022). Cu-Doped polypyrrole with multi-catalytic activities for Sono-Enhanced nanocatalytic tumor therapy. Small.

[17] Fu L-H, Hu Y-R, Qi C, He T, Jiang S, Jiang C, He J, Qu J, Lin J, Huang P (2019). Biodegradable manganese-doped calcium phosphate nanotheranostics for traceable cascade reaction-enhanced anti-tumor therapy. ACS Nano.

[18] Ranji-Burachaloo H, Gurr PA, Dunstan DE, Qiao GG (2018). Cancer treatment through nanoparticle-facilitated Fenton reaction. ACS Nano.

[19] Rodriguez R, Schreiber SL, Conrad M (2022). Persister cancer cells: iron addiction and vulnerability to ferroptosis. Mol Cell.

[20] Zhou A, Fang T, Chen K, Xu Y, Chen Z, Ning X (2022). Biomimetic activator of sonodynamic ferroptosis amplifies inherent peroxidation for improving the treatment of breast cancer. Small.

[21] Xu X-L, Zhang N-N, Shu G-F, Liu D, Qi J, Jin F-Y, Ji J-S, Du Y-Z (2021). A luminol-based self-illuminating nanocage as a reactive oxygen species amplifier to enhance deep tumor penetration and synergistic therapy. ACS Nano.

[22] Feng W, Shi W, Liu S, Liu H, Liu Y, Ge P, Zhang H (2021). Fe(III)-Shikonin supramolecular nanomedicine for combined therapy of tumor via ferroptosis and necroptosis. Adv Healthcare Mater.

[23] Bai J, Jia X, Zhen W, Cheng W, Jiang X (2017). A facile ion-doping strategy to regulate tumor microenvironments for enhanced multimodal tumor theranostics. J Am Chem Soc.

[24] Liu S, Zhang M, Jin H, Wang Z, Liu Y, Zhang S, Zhang H (2022). Iron-containing protein-mimic supramolecular iron delivery systems for ferroptosis tumor therapy. J Am Chem Soc.

[25] Sharma A, Das J (2019). Small molecules derived carbon dots: synthesis and applications in sensing, catalysis, imaging, and biomedicine. J Nanobiotechnology.

[26] Liu J, Li R, Yang B (2020). Carbon dots: a new type of carbon-based nanomaterial with wide applications. ACS Cent Sci.

[27] Wang B, Cai H, Waterhouse GIN, Qu X, Yang B, Lu S (2022). Carbon dots in bioimaging, biosensing and therapeutics: a comprehensive review. Small Sci.

[28] Yang M, Su B, Ma Z, Zheng X, Liu Y, Li Y, Ren J, Lu L, Yang B, Yu X (2023). Renal-friendly Li(+)-doped carbonized polymer dots activate Schwann cell autophagy for promoting peripheral nerve regeneration. Acta Biomater.

[29] Yu Y, Song M, Chen C, Du Y, Li C, Han Y, Yan F, Shi Z, Feng S (2020). Bortezomib-encapsulated CuS/carbon dot nanocomposites for enhanced photothermal therapy via stabilization of polyubiquitinated substrates in the proteasomal degradation pathway. ACS Nano.

[30] Pang W, Jiang P, Ding S, Bao Z, Wang N, Wang H, Qu J, Wang D, Gu B, Wei X (2020). Nucleolus-targeted photodynamic anticancer therapy using renal-clearable carbon dots. Adv Healthc Mater.

[31] Li D, Lin L, Fan Y, Liu L, Shen M, Wu R, Du L, Shi X (2021). Ultrasound-enhanced fluorescence imaging and chemotherapy of multidrug-resistant tumors using multifunctional dendrimer/carbon dot nanohybrids. Bioact Mater.

[32] Jiang Q, Liu L, Li Q, Cao Y, Chen D, Du Q, Yang X, Huang D, Pei R, Chen X, Huang G (2021). NIR-laser-triggered gadolinium-doped carbon dots for magnetic resonance imaging, drug delivery and combined photothermal chemotherapy for triple negative breast cancer. J Nanobiotechnol.

[33] Chen P, He X, Hu Y, Tian XL, Yu XQ, Zhang J (2023). Spleen-targeted mRNA delivery by amphiphilic carbon dots for tumor immunotherapy. ACS Appl Mater Interfaces.

[34] Yang M, Feng T, Chen Y, Liu J, Zhao X, Yang B (2020). Synchronously integration of Co, Fe dual-metal doping in Ru@C and CDs for boosted water splitting performances in alkaline media. Appl Catalysis B Environ.

[35] Yang M, Tang Q, Meng Y, Liu J, Feng T, Zhao X, Zhu S, Yu W, Yang B (2018). Reversible "Off-On" fluorescence of Zn(2+)-passivated carbon dots: mechanism and potential for the detection of EDTA and Zn(2). Langmuir.

[36] Tejwan N, Saini AK, Sharma A, Singh TA, Kumar N, Das J (2021). Metal-doped and hybrid carbon dots: a comprehensive review on their synthesis and biomedical applications. J Control Release.

[37] Liang W, Ferrara N (2020). Iron Metabolism in the tumor microenvironment: contributions of innate immune cells. Front Immunol.

[38] Kim SE, Zhang L, Ma K, Riegman M, Chen F, Ingold I, Conrad M, Turker MZ, Gao M, Jiang X (2016). Ultrasmall nanoparticles induce ferroptosis in nutrient-deprived cancer cells and suppress tumour growth. Nat Nanotechnol.

[39] Zanganeh S, Hutter G, Spitler R, Lenkov O, Mahmoudi M, Shaw A, Pajarinen JS, Nejadnik H, Goodman S, Moseley M (2016). Iron oxide nanoparticles inhibit tumour growth by inducing pro-inflammatory macrophage polarization in tumour tissues. Nat Nanotechnol.

[40] Ci Q, Wang Y, Wu B, Coy E, Li JJ, Jiang D, Zhang P, Wang G (2023). Fe-doped carbon dots as NIR-II fluorescence probe for in vivo gastric imaging and pH detection. Adv Sci (Weinh).

[41] Yang M, Meng Y, Liu J, Yu W, Yang B (2019). Facile synthesis of Mg2+ -doped carbon dots as novel biomaterial inducing cell osteoblastic differentiation. Part Part Syst Charact.

[42] Wang H, Zhang M, Ma Y, Wang B, Huang H, Liu Y, Shao M, Kang Z (2020). Carbon dots derived from citric acid and glutathione as a highly efficient intracellular reactive oxygen species scavenger for alleviating the lipopolysaccharide-induced inflammation in macrophages. ACS Appl Mater Interfaces.

[43] Liu M, Huang L, Xu X, Wei X, Yang X, Li X, Wang B, Xu Y, Li L, Yang Z (2022). Copper doped carbon dots for addressing bacterial biofilm formation, wound infection, and tooth staining. ACS Nano.

[44] Li X, Ding S, Lyu Z, Tieu P, Wang M, Feng Z, Pan X, Zhou Y, Niu X, Du D (2022). Single-atomic iron doped carbon dots with both photoluminescence and oxidase-like activity. Small.

[45] Liu J, Geng Y, Li D, Yao H, Huo Z, Li Y, Zhang K, Zhu S, Wei H, Xu W (2020). Deep red emissive carbonized polymer dots with unprecedented narrow full width at half maximum. Adv Mater.

[46] Du F, Zhang L, Zhang L, Zhang M, Gong A, Tan Y, Miao J, Gong Y, Sun M, Ju H (2017). Engineered gadolinium-doped carbon dots for magnetic resonance imaging-guided radiotherapy of tumors. Biomaterials.

[47] Yang F, Xiao Y, Ding JH, Jin X, Ma D, Li DQ, Shi JX, Huang W, Wang YP, Jiang YZ, Shao ZM (2023). Ferroptosis heterogeneity in triple-negative breast cancer reveals an innovative immunotherapy combination strategy. Cell Metab.

[48] Soares MP, Hamza I (2016). Macrophages and iron metabolism. Immunity.

[49] Ansari C, Tikhomirov GA, Hong SH, Falconer RA, Loadman PM, Gill JH, Castaneda R, Hazard FK, Tong L, Lenkov OD (2014). Development of novel tumor-targeted theranostic nanoparticles activated by membrane-type matrix metalloproteinases for combined cancer magnetic resonance imaging and therapy. Small.

[50] Li C (2014). A targeted approach to cancer imaging and therapy. Nat Mater.

[51] Li F, Rong Z, Zhang R, Niu S, Di X, Ni L, Liu C (2022). Vascular restenosis reduction with platelet membrane coated nanoparticle directed M2 macrophage polarization. iScience.

[52] Sun Y, Zuo Z, Kuang Y (2020). An Emerging target in the battle against osteoarthritis: macrophage polarization. Int J Mol Sci.

[53] DeNardo DG, Ruffell B (2019). Macrophages as regulators of tumour immunity and immunotherapy. Nat Rev Immunol.

[54] He C, Sun S, Zhang Y, Xie F, Li S (2021). The role of irreversible electroporation in promoting M1 macrophage polarization via regulating the HMGB1-RAGE-MAPK axis in pancreatic cancer. Oncoimmunology.

[55] Teng S, Li YE, Yang M, Qi R, Huang Y, Wang Q, Zhang Y, Chen S, Li S, Lin K (2020). Tissue-specific transcription reprogramming promotes liver metastasis of colorectal cancer. Cell Res.

[56] Choi HY, Ahn JH, Kwon H, Yim JH, Lee D, Choi JH (2022). Citromycin isolated from the antarctic marine-derived fungi, Sporothrix sp., inhibits ovarian cancer cell invasion via suppression of ERK signaling. Mar Drugs.

[57] Chen DW, Wang H, Bao YF, Xie K (2018). Notch signaling molecule is involved in the invasion of MiaPaCa2 cells induced by CoCl2 via regulating epithelial-mesenchymal transition. Mol Med Rep.

[58] Kang Y, Massague J (2004). Epithelial-mesenchymal transitions: twist in development and metastasis. Cell.

