# Supplementary material for: Fe-doped carbon dots: a novel biocompatible nanoplatform for multi-level cancer therapy
Source: J Nanobiotechnology. 2023 Nov 17;21:431. doi: 10.1186/s12951-023-02194-6 (PMC10655501; doi:10.1186/s12951-023-02194-6)
Supplement: Supplementary file 1 — Additional file 1: Additional figures S1–S25. [file 12951_2023_2194_MOESM1_ESM.docx]

Additional Information

**Fe-doped Carbon Dots: A Novel Biocompatible Nanoplatform for Multi-level Cancer Therapy**

Mingxi Yang^1,3^, Haiqiu Li^3^, Xinchen Liu^2^, Lei Huang^2^, Boya Zhang^2^, Kexuan Liu^2^, Wangni Xie^2^, Jing Cui^2^, Daowei Li^2^*, Laijin Lu^1,3^*, Honchen Sun^2^* and Bai Yang^1^*

* Corresponding Authors: Daowei Li, Laijin Lu, Honchen Sun, Bai Yang

[jluldw@jlu.edu.cn;](mailto:jluldw@jlu.edu.cn;) [lulaijin@hotmail.com](mailto:lulaijin@hotmail.com); [hcsun@jlu.edu.cn;](mailto:hcsun@jlu.edu.cn;) [byangchem@jlu.edu.cn](mailto:byangchem@jlu.edu.cn)

**Additional Figures**

**Figure S1.** EDS spectrum of Fe-CDs.

**Figure S2.** ICP spectra of Fe ions in Fe-CDs.

**Figure S3.** Photographs of solution color to evaluate the chromatic reaction of Fe^3+^ and SA. From left to right: SA+PBS, SA+FeSO_4_, Fe-CDs (2 mg/mL), SA+Fe-CDs (0.5 mg/mL), SA+Fe-CDs (2 mg/mL), and SA+Fe-CDs (4 mg/mL).

**Figure S4.** TEM images of Fe-CDs location in tumor cells ((a) ×10000 magnification, (b) ×50000 magnification).

**Figure S5.** MTT assay of human dental pulp stem cells (hDPSCs) incubated with different concentration of Fe-CDs at 1, 3, and 5d.

**Figure S6.** MTT assay of human umbilical vein cell fusion cells (EA.hy926) incubated with different concentration of Fe-CDs at 1, 3, and 5d.

**Figure S7.** (a) Effect of cell ferroptosis inhibitor (Fer), necrosis inhibitor (Nec), and apoptosis inhibitor (ZVAD) on the relative cell viability of tumor cells. (b) Effect of ZVAD on the cell viability at different inhibitor concentrations. (ns: not significant,* P<0.05, ** P<0.01, *** P<0.001, **** P<0.0001, mean±SD, n=6)

**Figure S8.** Gray value analysis of PARP, Caspase 9, Caspase 3, and BAX levels. (ns: not significant,* P<0.05, ** P<0.01, *** P<0.001, **** P<0.0001, mean±SD, n=6).

**Figure S9.** Comparison of cell viability of tumor cells incubated with G-CDs and Fe-CDs by MTT assay.

**Figure S10.** Cell apoptosis of tumor cells incubated with different concentration of G-CDs by flow cytometry.

**Figure S11.** Live/dead cell staining of tumor cells incubated with G-CDs (left) or Fe-CDs (right) at the concentration of 400 μg/mL for 24 h. (Scale bar=500 μm)

**Figure S12.** qPCR assay of IL-10 expression level in macrophages with tumor cell medium in the presence of G-CDs. (ns: not significant, **** P<0.0001, mean±SD, n=6)

**Figure S13.** Gray value analysis of Arg-1 levels in macrophages incubated with or without tumor cell medium in the presence (+) or absence (-) of FeG and Fe-CDs. (* P<0.05, **** P<0.0001, mean±SD, n=6).

**Figure S14.** Western blot and gray value analysis of expression levels of p-P38 in macrophages incubated with or without tumor cell medium in the presence (+) or absence (-) of FeG and Fe-CDs.(ns: not significant, **** P<0.0001, mean±SD, n=6).

**Figure S15.** Western blot of expression levels of M2-type macrophage-related proteins in macrophages treated with tumor cell medium without addition of Fe-CDs at different incubation time.

**Figure S16.** Gray value analysis of M2-type macrophage-related proteins levels in macrophages incubated with tumor cell medium without addition of Fe-CDs at different incubation time. (ns: not significant,* P<0.05, ** P<0.01, *** P<0.001, **** P<0.0001, mean±SD, n=6).

**Figure S17.** Gray value analysis of M2-type macrophage-related proteins levels in macrophages incubated with tumor cell medium with addition of Fe-CDs at different incubation time. (ns: not significant,* P<0.05, ** P<0.01, *** P<0.001, **** P<0.0001, mean±SD, n=6).

**Figure S18.** (a) Scratch test of the effect of PBS and Fe-CDs on the migration ability of tumor cells (Scale bar=200 μm). (b) Transwell assay (left) (Scale bar=200 μm) and cell morphology images (right) (Scale bar=100 μm) of the effect of PBS and Fe-CDs on the migration ability of tumor cells.

**Figure S19.** Migration cell count from transwell experiments of control, Fe-CDs, CoCl_2_ and CoCl_2_+Fe-CDs groups.(** P<0.01, **** P<0.0001, mean±SD, n=6).

**Figure S20.** Scratch test of the effect of G-CDs and Fe-CDs on the migration ability of tumor cells at 24 h. (Scale bar=500 μm)

**Figure S21.** Western blot of expression levels of EMT-related proteins in tumor cells treated with different concentration of Fe-CDs at 3 d.

**Figure S22.** Gray value analysis of EMT-related protein levels in tumor cells incubated with different concentration of Fe-CDs at 3 d. (ns: not significant,* P<0.05, ** P<0.01, *** P<0.001, **** P<0.0001, mean±SD, n=6).

**Figure S23.** Western blot of expression levels of EMT-related proteins in tumor cells treated with CoCl_2_ at different incubation time.

**Figure S24.** Gray value analysis of EMT-related proteins levels in tumor cells incubated with CoCl_2_ at different incubation time. (ns: not significant,* P<0.05, ** P<0.01, *** P<0.001, **** P<0.0001, mean±SD, n=6).

**Figure S25.** Gray value analysis of EMT-related proteins levels in tumor cells incubated with CoCl_2_ and Fe-CDs at different incubation time. (ns: not significant,* P<0.05, ** P<0.01, *** P<0.001, **** P<0.0001, mean±SD, n=6).
